# How to Arrive at Estimates

**Published:** 1907-06-29

**Authors:** 


### Nursing Administration.

### HOW TO ARRIVE AT ESTIMATES.

The matron who is once thoroughly convinced of the importance of making and keeping within precise estimates of housekeeping expenditure under separate headings, will deem no pains lost which result in giving her this power over the outgoings and incomings. But those who have never made the attempt are apt greatly to exaggerate in their own minds the labour of keeping distinct the cost of the different sections of the institution. The principal requisites are a clear system of accounts, extending to the form in which the tradesmen tender their weekly accounts, and separate larders, or, if this be not possible, a larder capable of being divided into different sections for the reception of provisions for household and patients. The manner in which it works out is as follows. The housekeeper under this system devotes an hour in the early afternoon, after the kitchen dinner is over and all has been tidied up, to making up her requisitions for the ensuing twenty-four hours. She has first to consider what is needed. For the patients there is little difficulty in deciding. The diet sheets have been sent down from the different wards, and the amount of milk, beef tea, fish, meat, vegetables, pudding, etc., required for the night and day is clearly entered on each. A brief calculation places her in possession of the necessary quantity of rice, of sugar, of eggs for puddings, etc., which will have to be issued to the cook on this account, and the amount of tea and other items needed by each sister for use in the ward kitchen. She can tell to a fraction exactly what the patients are going to consume. Next there is the household, and in hospitals where there is not a large medical staff, it will be sufficient to deal with the household requisitions under one heading. What meals will be required? In a hospital of moderate size the following tables will have to be provided for in the twenty-four hours:—

Breakfast for officers, nurses, servants; dinner for night nurses; standing lunches as required; dinner for nurses, servants; luncheon for officers; tea for officers, nurses, servants; dinner for officers; supper for nurses, servants; rations for night nurses.

The housekeeper will have a list of the numbers to be provided for at each meal, and taking them in detail will make her own requisition of the amounts required under the headings supplied in the Uniform System of Accounts. For such items as bread and milk she will count heads and estimate, according to experience, the quantity needed. The cook's assistance will be needed to estimate the quantities, say, for the stewed fruit and custard which is to appear at the officers' dinner, or for the dish of macaroni and cheese which is for the nurses' supper. In estimating the quantities of meat, she will reckon half a pound of uncooked meat a head. Not that this will by any means always be needed, but when bone and waste in cooking are taken into count, it is not safe to provide on a smaller scale.

Finally, when she has formed a clear estimate of her needs for the following day, and combined them all under the headings of Patients and Household on the requisition sheet, she will proceed to make out her orders. There are three sources of supply. First there is the contents of the larders. These she will inspect with the cook, and select from the remains of the dinner just ended whatever can be used for the suppers. For instance, if the requisition sheet shows that some forty people have to be provided with a meat supper, she will compute the weight of what is left, and supplement it as required. A clever cook will often be able with half a leg of mutton to compound a savoury stew with a few tomatoes and some spaghetti or rice, which will make a delicious supper for a large party, far more acceptable than a slab of cold meat, and at half the cost. When the cook understands that glory will reflect upon her if the week's average is satisfactory, and that everyone is puzzled and sorry when the figure runs up, she will prove very fertile in inventing all sorts of expedients for using up the remains.

The second source of supply is the tradesman or contractor who delivers each day such perishable provisions as are needed. For each of these a signed order has to be made out, the counterfoils remaining in her own possession showing how much is for patients and how much for household.

Thirdly, the same process is to be gone through on a separate set of orders on the store-room for provisions kept in stock. These she will give out herself in the morning, but, none the less, she will file the orders and keep them for reference.

The housekeeper's daily requisition sheet, if this order of procedure be followed, shows the exact amount of everything consumed in the institution, under a double entry. To estimate the weekly cost of board, it is merely necessary to turn each item into money, and this is a question of a few hours' clerical work. In order to facilitate it, the tradesmen—supposing that weekly accounts are rendered, as they ought to be—should be provided with books ruled in such a way that the quantity and cost of any given article supplied in the week can be seen at a glance.

The regularity demanded by this system does far more than furnish information with regard to the expenditure. It keeps the whole domestic staff on the alert, and the knowledge it imparts concerning the quantities used in the household has the highest educational effect on the housekeeper herself. It is, in fact, an admirable training system, and when it is substituted for the common guesswork and rule of thumb management, it imparts an entirely new element of intelligent interest into duties which, through long routine, are liable to appear dull and unimportant.

